# Systematic inspection on the interplay between MoNa-induced sodium and the formation of MoSe_2_ intermediate layer in CIGSe/Mo heterostructures

**DOI:** 10.1007/s11356-024-32938-2

**Published:** 2024-03-21

**Authors:** Fazliyana ‘Izzati Za’abar, Camellia Doroody, Manzoore Elahi Mohammad Soudagar, Puvaneswaran Chelvanathan, Wan Syakirah Wan Abdullah, Ahmad Wafi Mahmood Zuhd, Erdem Cuce, Shaik Saboor

**Affiliations:** 1 UNITEN R&D Sdn. Bhd., Universiti Tenaga Nasional (UNITEN), 43000 Kajang, Selangor Malaysia; 2 Institute of Sustainable Energy, Universiti Tenaga Nasional (UNITEN), 43000 Kajang, Selangor Malaysia; 3 Faculty of Mechanical Engineering, Opole University of Technology, 45-758 Opole, Poland; 4 Department of Mechanical Engineering, Graphic Era (Deemed to be University), Dehradun, Uttarakhand 248002 India; 5 Solar Energy Research Institute (SERI), Universiti Kebangsaan Malaysia (UKM), 43600 Bangi, Selangor Malaysia; 6 TNB Renewables Sdn. BhdPJX HM-Shah TowerTenaga Nasional Berhad, Level 31, Kuala Lumpur, Malaysia; 7 Department of Mechanical Engineering, Faculty of Engineering and Architecture, Recep Tayyip Erdogan University, Zihni Derin Campus, 53100 Rize, Turkey; 8 School of Engineering and the Built Environment, Birmingham City University, Birmingham, B4 7XG UK; 9 School of Mechanical Engineering, Vellore Institute of Technology, Vellore, 632014 Tamil Nadu India

**Keywords:** Energy, CIGSe, Solar cells, MoNa, Sputtering, Molybdenum, MoSe_2_, Sodium

## Abstract

The critical impact of sodium-doped molybdenum (MoNa) in shaping the MoSe_2_ interfacial layer, influencing the electrical properties of CIGSe/Mo heterostructures, and achieving optimal MoSe_2_ formation conditions, leading to improved hetero-contact quality. Notably, samples with a 600-nm-thick MoNa layer demonstrate the highest resistivity (73 μΩcm) and sheet resistance (0.45 Ω/square), highlighting the substantial impact of MoNa layer thickness on electrical conductivity. Controlled sodium diffusion through MoNa layers is essential for achieving desirable electrical characteristics, influencing Na diffusion rates, grain sizes, and overall morphology, as elucidated by EDX and FESEM analyses. Additionally, XRD results provide insights into the spontaneous peeling-off phenomenon, with the sample featuring a ~ 600-nm MoNa layer displaying the strongest diffraction peak and the largest crystal size, indicative of enhanced Mo to MoSe_2_ conversion facilitated by sodium presence. Raman spectra further confirm the presence of MoSe_2_, with its thickness correlating with MoNa layer thickness. The observed increase in resistance and decrease in conductivity with rising MoSe_2_ layer thickness underscore the critical importance of optimal MoSe_2_ formation for transitioning from Schottky to ohmic contact in CIGSe/Mo heterostructures. Ultimately, significant factors to the advancement of CIGSe thin-film solar cell production are discussed, providing nuanced insights into the interplay of MoNa and MoSe_2_, elucidating their collective impact on the electrical characteristics of CIGSe/Mo heterostructures.

**Graphical Abstract:**

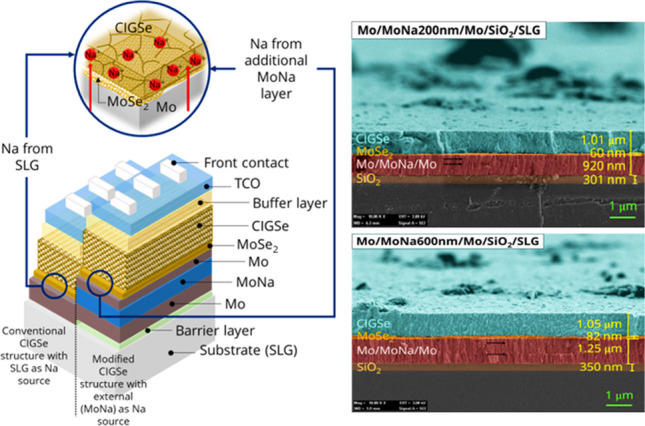

## Introduction

Thin-film solar cell technology has attracted the interest of research owing to its low cost, sustainability, and high efficiency, making thin films a promising option for long-term electricity production (Cuce 2014). Thin film solar cells are also outstanding in terms of enhanced solar cell parameters compared to conventional Si photovoltaic cells (Cuce and Bali 2009). Recently, copper indium gallium selenide (CIGSe) solar cells achieved a new record of 23.35% effective performance (Nakamura et al. 2019; Salhi 2022). Recent developments in CIGSe solar cells have focused on improving efficiency, stability, and manufacturing processes. There is a growing interest in flexible CIGSe solar panels, which are known for their high efficiency, albeit at a relatively higher cost compared to other types of solar cells. However, there is ongoing challenges related to the back contact structure and sodium (Na) incorporation in flexible CIGSe solar cells (Mufti et al. 2020; Regmi et al. 2020). To develop optimal CIGSe devices, a highly conductive back contact is required to facilitate efficient charge carrier extraction (Doroody et al. 2019). Mo emerges as the dominant choice for back contact in CIGSe solar cells due to its relative stability at the processing temperature, resistance to alloying with Cu and In, and its low contact resistance to CIGSe (Ramanujam and Singh 2017; Wang et al. 2013). During the high-temperature growth of the CIGSe absorber layer, a thin layer of molybdenum diselenide (MoSe_2_) is formed at the interface between Mo and CIGSe, due to the selenization or selenium diffusion into the Mo. This inter-layer is particularly efficient in its ideal thickness to minimize contact resistance at the CIGSe/Mo junction, and reduced reliability (Abou-Ras et al. 2005a; Hsiao et al. 2013; Klinkert et al. 2016; Lee et al. 2013; Lin et al. 2014a, b, c; Lin et al. 2014a, b, c; Salhi 2022; Sun et al. 2019; Za’abar et al. 2023). While the MoSe_2_ layer is considered to be crucial for achieving high efficiency in CIGSe solar cells, excessive thickness leads to reduced electrical characteristics of the cell due to the high resistivity of MoSe_2_ and potential delamination issues (Gouillart et al. 2021). It has been reported that various parameters can impact the growth of the MoSe_2_ layer, particularly the physical properties of the Mo film, such as compactness, stress, and grain orientation, which are highly dependent on the deposition method and growth recipe of the film (Daniel Abou-Ras et al. 2005a, b, c; Lin et al. 2014a, b, c; Liu et al. 2018; Zhu et al. 2012a). Therefore, gaining insights into the formation mechanism of the MoSe_2_ layer and optimizing the subsequent Mo deposition process are crucial for developing CIGSe cells with higher efficiency and a robust manufacturing process. By understanding the factors influencing MoSe_2_ growth and optimizing the Mo deposition, the aim is to overcome the limitations associated with excessive MoSe_2_ thickness and improve the overall performance of CIGSe solar cells.

The supply of Na in CIGSe solar cells presents a significant challenge and has a direct impact on their performance and efficiency. The effective concentration of Na diffusion has been demonstrated to be a performance-enhancing element in the CIGSe device through the back contact characteristic improvement (Lin et al. 2014a, b, c; Ong et al. 2018; Ramanujam and Singh 2017). In conventional CIGSe solar cells, Na naturally diffuses from soda-lime glass (SLG) substrate into the CIGSe absorber layer during the high temperature growth of the absorber (Bhatt et al. 2023). However, controlled Na diffusion from the substrate has been proven challenging in these cases. To address this issue, several research groups have explored the application of Na source materials, such as NaF, Na_2_Se, and Na-doped molybdenum (Mo) known as MoNa along with an alkali barrier to regulate Na diffusion from the substrate (Blösch et al. 2013a, b; Lin et al. 2016; Mansfield et al. 2011; Ramanujam and Singh 2017; Reinhard et al. 2015; Wang et al. 2017; Wuerz et al. 2011). The deliberate incorporation of MoNa within the back contact design of a sandwich stack configuration has been reported by Blösch et al. (2014), Blösch et al. (2013a, b), Blösch et al. (2013a, b), and Yoon et al. (2010). With the introduction of MoNa replacing SLG, better control of Na diffusion can be achieved, which is said to influence the formation of the MoSe_2_ interfacial layer, in CIGSe/Mo heterostructure. The deficits of excessive Na diffusion at the CIGSe/Mo junction is correlated to the shift in the miller indices and the MoSe_2_ c-axis crystallite intersecting from parallel to the substrate to perpendicular, increasing the anisotropic nature of MoSe_2_ and the electrical resistivity of the final device.

According to a study by Yoon et al. (2014), the incorporation of Na reduces contact resistance by enhancing the Na doping of CIGSe and MoSe_2_. In general, two potential effects of Na on the MoSe_2_ thin film that are significant are reported: first, Na influence on the MoSe_2_ layer appearance, properties, and thickness, and second, the resistivity of inevitably formed MoSe_2_ layer, which directly relates to Na doping ratio and needs a precise control to avoid high series resistance. In a recent report (Abou-Ras et al. 2005b), the MoSe_2_ layer formation dynamics are analyzed while an extreme interlayer thickness increment with increasing the CIGSe deposition temperature is presented. Zhu et al. (2012b) inspected the Na dopant ratio impact on the MoSe_2_ layer formation pace, particularly by introducing Mo electrodes with varied density as well as controlling Na diffusion from the glass substrate into the Mo with a thin SiO_2_ coating at the glass/Mo interface. It proved that the device’s excessive series resistance was caused by an overly thick MoSe_2_ layer. There was also a considerable decrease in efficiency reported in that report due to the absorber layer peeling off from the Mo layer when the MoSe_2_ layer was excessively thin.

To date, numerous studies have been published focusing on the incorporation of Na using MoNa coating. Respectively, these investigations have delved into the impacts of MoNa layer thickness and composition (Na content) (Blösch et al. 2014), as well as the influence of the CIGSe deposition temperature (Salomé et al. 2013) on both the growth of CIGSe and the performance of solar cells. However, the influence of MoNa layer insertion on the formation of the MoSe_2_ phase and its impact on cell performance is yet to be well understood. While previous research has extensively studied CIGSe solar cells, this work distinguishes itself through its targeted investigation into the optimization and tailored inclusion of Na through MoNa layer incorporation, to enhance the interface between CIGSe and Mo, that is critical for overall solar cell performance. The core objective of this study is to assess the relationship between the MoNa layer, the formation of the MoSe_2_ compound, and its associated characteristics in CIGSe/Mo heterostructure. In particular, the influence of the MoNa layer on the MoSe_2_ growth rate and thickness is analyzed, and the impact of controlled Na diffusion through MoNa layer on the electrical properties of the CIGSe/Mo heterostructures is addressed. The findings of this study contribute to the advancement of back contact optimization by providing novel insights into the function and potential of MoNa as the Na source, which is particularly beneficial for the development of flexible CIGSe solar cells.

## Methodology

**Fig. 1:**
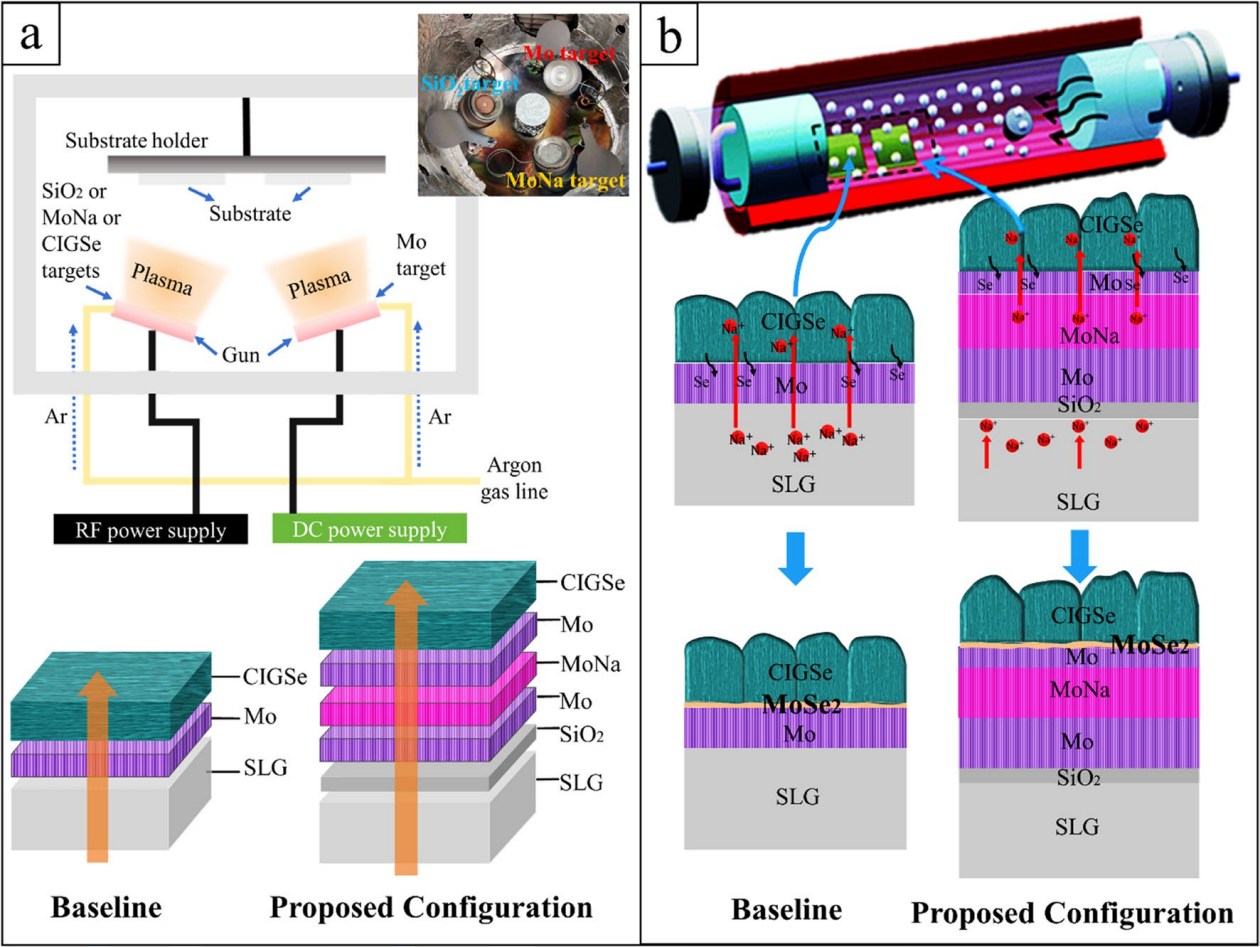
**a** Sequential sputtering of SiO_2_, Mo, MoNa, and CIGSe layers; **b** annealing in Se-free environment

In this study, a sandwich stack configuration of Mo/MoNa/Mo thin films is proposed. MoNa film was grown by a Mo-10% Na on SiO_2_-coated soda-lime glass (SLG) substrate of 3 × 3 × 1.1 cm^3^. The Na atomic concentration in the used target was 10%; this Na was added to the target as Na_2_MoO_4_ compound (Plansee Pte. Ltd). Mo layers were sputtered before and after MoNa film deposition to a thickness ranging of 400 nm and 200 nm, respectively, at 100 W power, a work pressure of 5 × 10^−3^ Torr, 16 sccm dynamic argon (Ar) flow, and substrate temperature of 100 °C (Za’abar et al. 2023). A single Mo layer on SLG without a MoNa layer was used for reference. Various Mo-10%Na films were prepared with thicknesses ranging from 200 to 600 nm which translates to a sputtering time of 60 to 180 min, under a working pressure of 3 × 10^−3^ Torr, and a power of 100W, at a process temperature of 300 °C. Because of the comparatively small working pressure, a high mean free path formed, enabling an effective sputtering rate onto the samples (Zhou et al. 2016). The sample holder’s spin speed and the distance from the target to samples were adjusted at 1 rpm and 110 mm. Under these sputter coating settings, thin films with thickness differences of less than 6% across SLG substrates were evenly coated. Figure 1a depicts the chronological sputter deposition and the chamber condition. CIGSe precursor absorber layers were sputtered on the Mo films (CIGSe/Mo films) from a quaternary CIGSe target under the same conditions for all the prepared samples, and a 1-µm-thick CIGSe absorber layer was developed. Annealing of the CIGSe samples was then processed in a quartz tube chamber in a Se-free environment with N_2_ (purity 99.9999%), demonstrated in Fig. 1b.

**Fig. 2:**
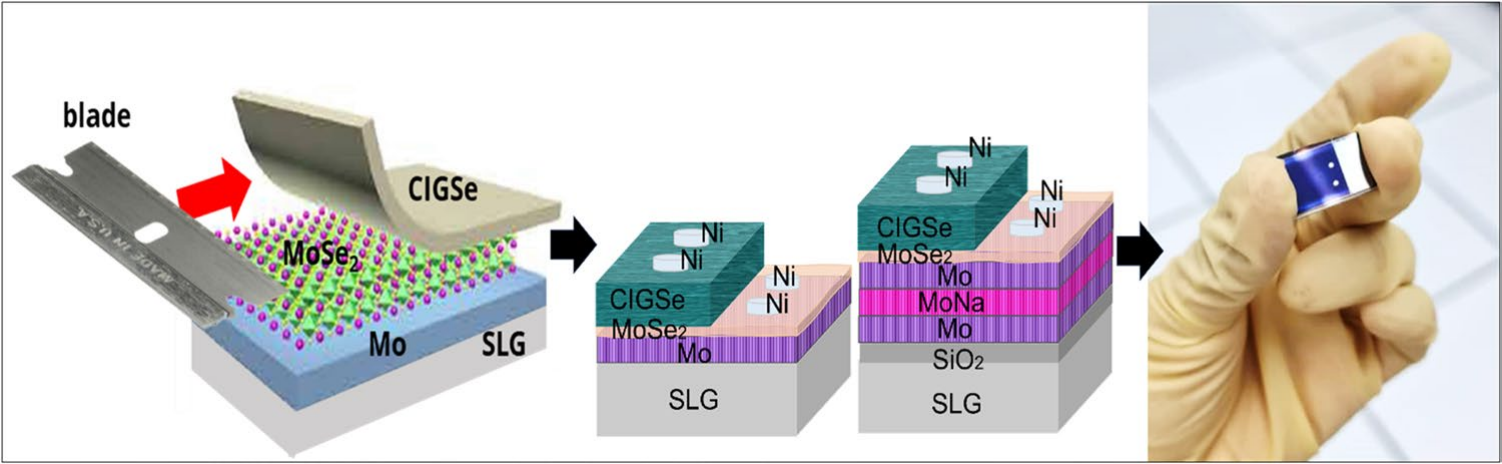
Mechanical exfoliation of CIGSe thin film and Ni front contact deposition

In line with the investigations conducted by Abou-Ras et al. study (Abou-Ras et al. 2005a), heat treatment at 540 to 550 °C was applied in a Se-free environment for 30 min, and it was observed that the thickness of MoSe_2_ remained relatively low when the substrate temperature is below 550 °C during the selenization process. However, a rapid escalation in thickness started when the temperature exceeded 550 °C. Receiving heat treatment, the generated stack appeared to be comprised of four layers: CIGSe/MoSe_2_/Mo/SLG. The detachment phenomenon can be anticipated in 2D semiconductors with weak van der Walls linkages with hosting holders, such as the MoSe_2_, as addressed in prior works (Weinhardt et al. 2006, 2007). Following the lift-off as illustrated in Fig. 2, two surfaces of the CIGSe and exposed Mo layer can be differentiated, enabling a thorough analysis of the CIGSe/Mo interface. As demonstrated in Fig. 2 contact layer of 100-nm Ni was deposited as electrode on Mo and CIGSe surfaces using electron beam evaporation to examine the electrical capabilities of the Mo electrode/CIGSe.

Lift-off applications must maintain quality while having no detrimental influence on structural and/or electrical properties. The mechanical approach is particularly relevant among the two known lift-off processes, chemical and mechanical, because cleavage is relatively easy along weak van der Waals planes (Klinkert et al. 2016). For each sample, two surfaces can simply be distinguished upon lift-off: the top surface of the CIGSe absorber layer and the exposed MoSe_2_/(Mo/MoNa/Mo) side of the back contact. A Bruker Dektak stylus analyzer was used to quantify the thickness of samples. The structural parameters and crystallinity across the Mo and CIGSe/Mo films with and without a MoNa layer were evaluated by X-ray powder diffraction (XRPD) of BRUKER AXS D8 Advance Cu-Kα diffractometer in 2*θ* range from 20 to 80°, with 0.02 count and *λ* = 1.5408 Å. CARL ZEISS MERLIN Field Emission Microscopy (FESEM) at 3 kV was used to acquire morphological properties in top and cross-sectional views, while the 5-μm scan size using NX-10 Park System was targeted. Electrical parameters were calculated by Ecopia HMS 3000 Hall Effect system with a magnetic field of 0.57 T. Raman scattering was measured by Renishaw InVia confocal microscope equipped with a charge-coupled device detector. The present research employed an emission spectrum and HeCd laser power of 532 nm and 10 mW. Dark I-V curves were assessed ultimately to reveal the diode characteristics at standard environment.

## Results and discussion

To highlight the optimal Na content, Na-doped Mo (MoNa) coating as an alternative Na source was compared to the conventionally used SLG substrate. The respective sputter time of the MoNa target was varied. Schematic designs of the CIGSe/Mo heterostructures and the measured thickness of individual MoNa layer and Mo/MoNa/Mo stack in each sample are presented in Table 1. By modulating the thickness of the MoNa layer, Na concentration in Mo-based back contact is systematically controlled to gain insights into Na diffusion impact on the growth of the MoSe_2_ interlayer. Sample nomenclature and measured film thickness using a DEKTAK profilometer are illustrated.

**Table 1:**
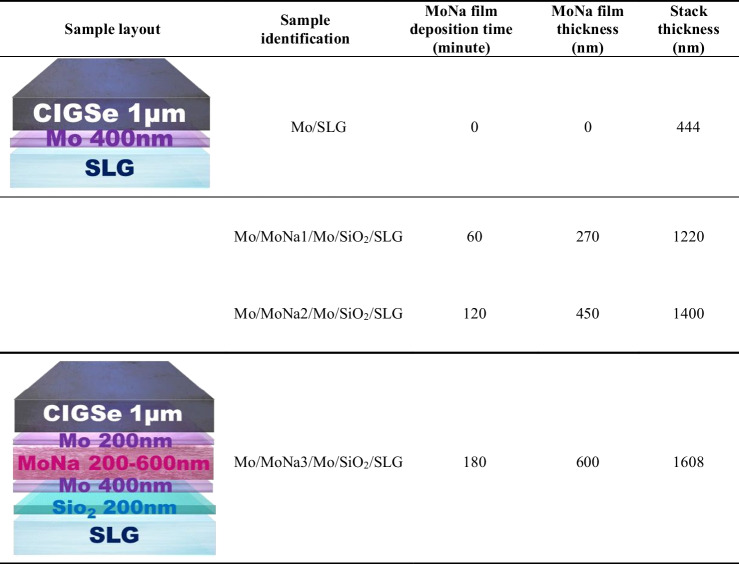
Na dopant alteration with variation of the sputter times (translated into variation in thickness) for the MoNa target

**Fig. 3:**
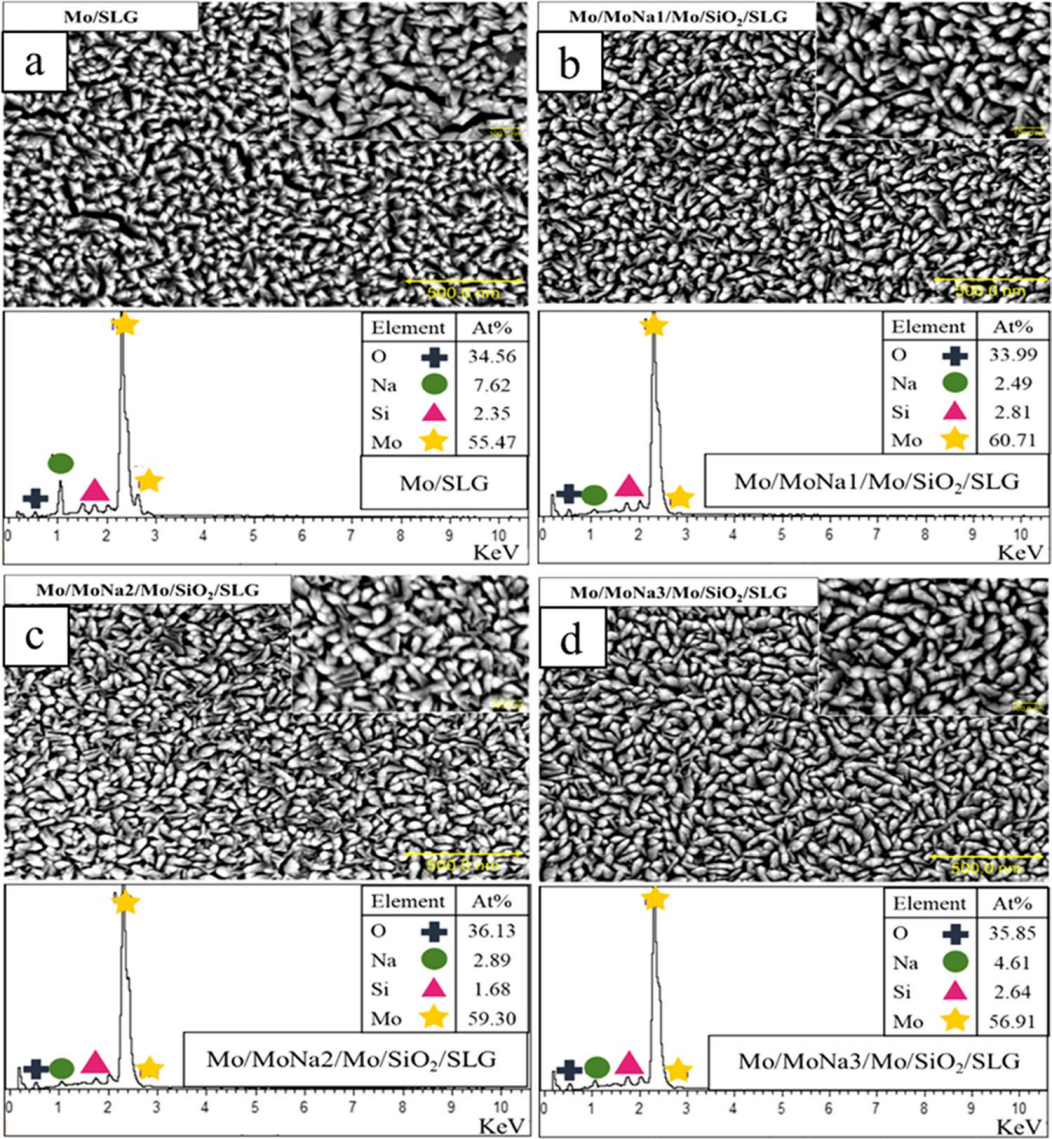
(Top) FESEM images showing the surface morphology and (Bottom) EDX graphs demonstrate composition of sputtered Mo/MoNa/Mo films with different thickness of MoNa: **a** 0 nm, **b** 200 nm, **c** 400 nm, and **d** 600 nm

Consequently, morphological and compositional characteristics of the Mo surface that caps the MoNa layer is studied. Figure 3 displays the EDX and FESEM images where in the case of Mo samples without MoNa layer exhibits the highest Na content and the Mo grains exhibit pyramidal appearance or cone-shaped structures. In the absence of the MoNa layer, the baseline leads to an additional porous morphology exhibiting prominent holes or inter-grain voids. The highest atomic percentage (at.%) of Na at 7.62 was observed in the reference sample, where SLG serves as the primary source of Na. Likewise, by MoNa layer thickness increases from an average of 200 to 600 nm, Na diffusion rate on the surface of Mo escalated from 2.49 to 4.61 at.%. Although these values are within the range of the sputtering target content (10 at.%), they remain comparatively lower. This is because the Na content in the sputtered MoNa film may differ from the target due to the sputter system’s atmosphere and construction. Plansee estimates that the film’s Na percentage is only 1–1.5 at.%, even with a MoNa target containing 3 at.% of Na. Thereby, for the 10 at.% of Na in the MoNa target used in this study, it is assumed that the MoNa layer consists of roughly 3.5–5 at.% of Na. On the other hand, the reduced amount of Na diffused from SLG through Mo layer of ~ 400 nm and from MoNa through Mo cap layer of ~ 200 nm could be attributed to the effectiveness of Na transport through the microstructure of Mo layer. The extent of Na diffusion from the SLG substrate and MoNa film varies significantly, influenced by both the Na source’s uniformity and quality, as well as the Mo back electrode’s capacity for Na transport. Table 2 summarizes the comparison in the variation of the at.% of Na in the source, in the MoNa layer, and in Mo layer.

**Table 2:** Na doping concentration variation in the Na source and in Mo layer

Sample identification	MoNa film thickness (nm)	Na source/Na at.% in Na source (%)	Estimated Na at.% in MoNa layer (%)	Na at.% in Mo cap layer (%)
Mo/SLG	0	SLG/13	-	7.62
Mo/MoNa1/Mo/SiO_2_/SLG	270	MoNa/10	3.5–5	2.49
Mo/MoNa2/Mo/SiO_2_/SLG	450			2.89
Mo/MoNa3/Mo/SiO_2_/SLG	600			4.61

**Fig. 4:**
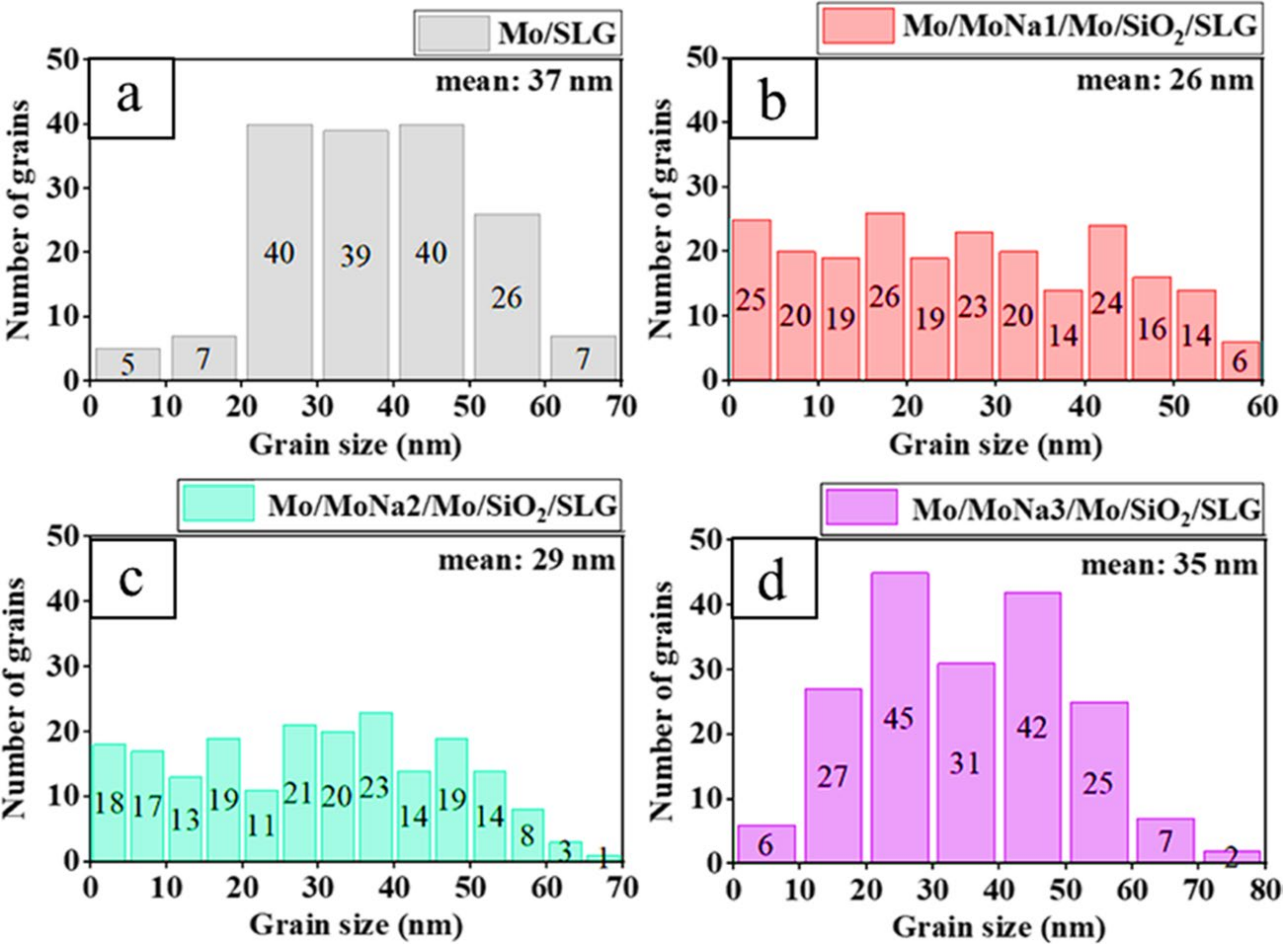
Distribution of Mo grain size with thickness variations of MoNa films

Figure 4 depicts the grain distribution resulting from FESEM top view data as bar graphs. Grain sizes are found to be in the range of less than 10 to 80 nm in length, with a median grains of 26 nm, 29 nm, 35 nm, and 37 nm for samples with MoNa layer thickness of ~ 200 nm, ~ 400 nm, ~ 600 nm, and baseline without MoNa layer, respectively. In relation to the morphology variation in Mo films, and the increase of diffused Na content, the grain size of the Mo cap layer increases. The findings here are relatable to other recent reports (Klinkert et al. 2016; Weinhardt et al. 2006, 2007; Sung et al. 2015). This effect of Na on the grain growth of Mo film is comparable to the enhancement of CIGSe grain size with Na incorporation either from the glass substrate or from external Na source as documented in (Li et al. 2019).

**Fig. 5:**
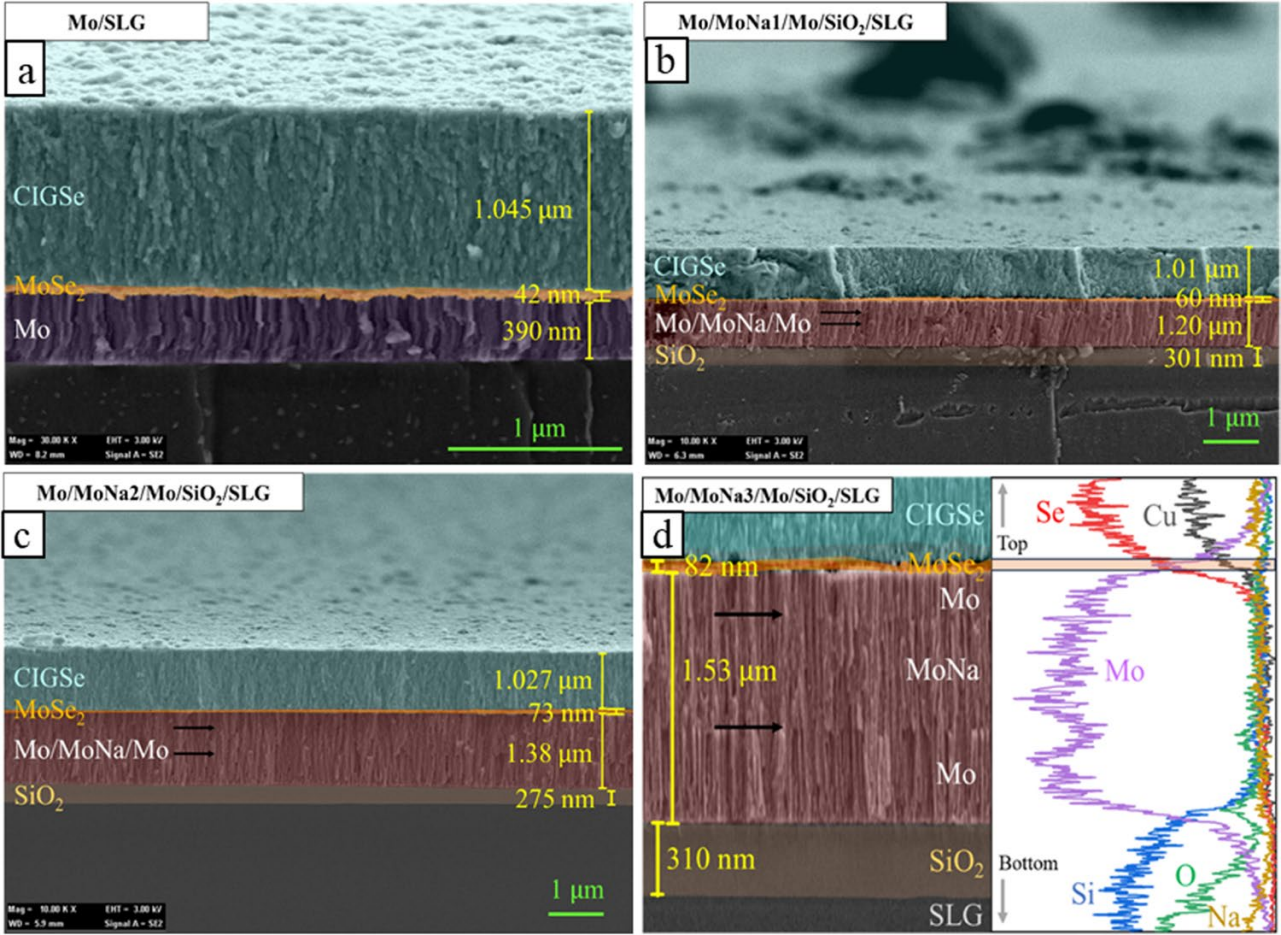
Cross-sectional FESEM images of the CIGSe grown on Mo **a** without and with MoNa of **b** ~ 200 nm, **c** ~ 400 nm, and **d** ~ 600 nm thick showing the presence of MoSe_2_ after annealing in Se-free atmosphere

To highlight the MoNa thickness variations on the formation of MoSe_2_, all Mo/MoNa/Mo films prepared earlier underwent CIGSe precursor deposition by sputtering followed by heat treatment in a Se-free environment. FESEM cross-sectional views of samples with various MoNa thickness as shown in Fig. 5 were adopted for calculating the thickness of interlayers such as MoSe_2_ and Mo thin films. Even though the surface of Mo films in the samples with and without MoNa layer displays slightly different surface structures as illustrated earlier in Fig. 3, their cross sections show nearly similar columnar patterns perpendicular to the substrate planes. In contrast, according to Sung et al. (Sung et al. 2015), the Mo layer has an average cylindrical form, but the MoNa layer features fibers with significantly reduced widths, implying that Na faces restrictions in grain formation when diffuse in MoNa layer.

**Fig. 6:**
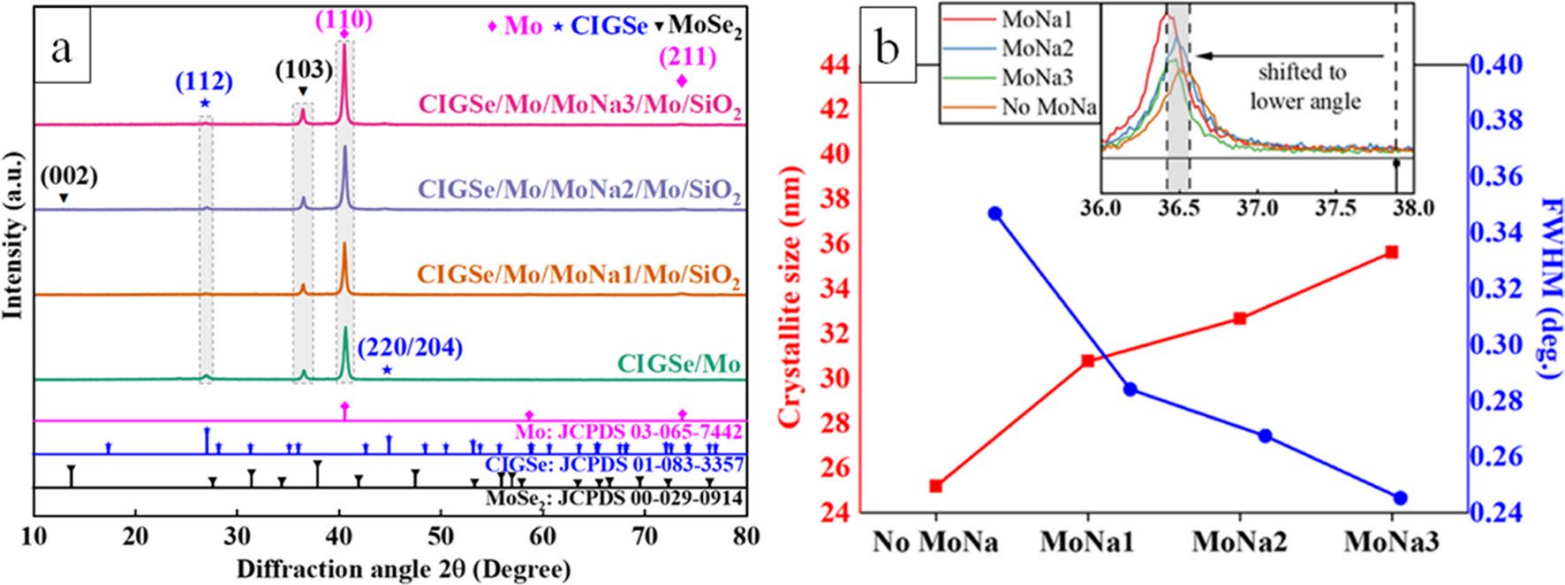
**a** 2D XRD graph of CIGSe/Mo samples deposited via varying Mo deposition power. **b** Crystal characteristics derived from the XRD analysis for different samples

EDX tests indicate the bottom coating as Mo with Na and the upper coating as MoSe_2_, and the separation in three layers of Mo/MoNa/Mo is indicated by the black arrows. Thinner than 100 nm of MoSe_2_ layer is formed at CIGSe/Mo interface in all samples by CIGSe growth and annealing in Se-free chamber. Cross-section figures display the separation in three layers of Mo/MoNa/Mo and a clear growth trend of the MoSe_2_ layer. EDX chemical components line mapping shown in Fig. 5d confirms the identity of the observed layer as MoSe_2_. The thickness of the MoSe_2_ interlayer increased by more than 37% from 60 to 82 nm with an increment in the thickness of the MoNa layer from 200 to 600 nm. Nevertheless, MoSe_2_ thickness was the lowest with an average value of 42 nm in the baseline structure. As shown in Fig. 3 earlier, Na concentration at the Mo surface was the highest when SLG served as the main Na source. The following explains why excessive and direct Na diffusion from the SLG limits the thickness of the MoSe_2_ coating. This study proposes three possible factors that may contribute to this phenomenon: (1) Grain density rises as Na dopant concentration rises, but Se vapor transfer via CIGSe falls as grain boundaries improve (Wang et al. 2019). (2) Na ions passivate surface and boundary defects in CIGSe, limiting Se atom diffusion (Yu et al. 2021). (3) The development of Na_2_Se_x_ compound on the surface and at grain boundaries in CIGSe serves as an inhibitor of diffusion to Se vapor (Li et al. 2019). According to latest research, MoSe_2_ layer thickness has a significant impact on the electrical performance of the resultant CIGSe device (Abou-Ras et al. 2005c), especially if MoSe_2_ becomes extremely thick. An acceptable thickness for the MoSe_2_ layer which is less than 100 nm favors the chalcopyrite CIGSe formation (Malik et al. 2015). Figure 6a shows the crystallite pattern of the CIGSe/Mo samples with and without the MoNa layer within 10 to 80°. The major Mo (110) plane at 2*θ* = 40.44° presenting monocrystalline qualities of the sputtered Mo films, described for low pressure film growth, was common to all the films (Rashid et al. 2019). The obtained data are classified in JCPDS card No. 65–7442 category for the Mo thin film crystallizes in body-centered cubic (BCC) structure.

The alteration in the MoNa thickness layer caused no significant movement in the peak position as shown in Fig. 6a. The resulting reduction in the metallic-Mo phases was predictable since the MoSe_2_ compound growth rate increase as an effect of Na diffusion (Lin et al. 2014a, b, c). In all samples, CIGSe films treated in a Se-free atmosphere to appear to have a chalcopyrite structure (JCPDS number 83–3355). The pick intensity of the CIGSe phase at 2*θ* = 27° corresponding to the (112) reflection plane is slightly higher in the sample without the MoNa layer. There is no secondary phase detected, which offers good crystallinity and a high phase purity for the annealed CIGSe films. The weak CIGSe peak in the XRD spectra of all samples can be attributed to the spontaneous peeling off of the CIGSe layer after high-temperature Se-free annealing (Hexin et al. 2011). The peeling off of the CIGSe layer can be caused by factors such as the GaSe growth at the CIGSe/Mo junction (Fleutot et al. 2014) and a thick MoSe_2_ layer that is not oriented parallel to the substrate [Patent: KR20140068306A]. As determined by structural analysis, the MoSe_2_ layer is formed, and its relative peaks are plotted in XRD graph. The MoSe_2_ compound formed at the CIGSe/Mo during the sputtering of CIGSe was identical to those of MoSe_2_, with hexagonal unit cells corresponding to JCPDS card number 29–0914. For hexagonal MoSe_2_, the crystal orientation can be controlled depending on the substrate and growth condition, and the controllable crystal orientation in MoSe_2_ samples can correspond to the (002), (004), (100), (101), (103), (006) planes (Balati et al. 2019; Liu et al. 2018). According to (Bougouma et al. 2008), for B-type MoSe_2_, with the strong diffracted points at (002), (100), (103), and (105) planes, the XRD pattern at (00 l) basal plane is not highlighted. The crystal structure of MoSe_2_ is two-dimensional and consists of two MoSe_2_ sheets stacked together with weak atomic bonds. The MoSe_2_ (100) and (110) peaks correspond to perpendicular planes, the (002) peak to parallel planes to the substrate, and the (103) peak to tilted structure towards the substrate. (Gao et al. 2018). In the figure, only the (103) peak of MoSe_2_ at 2*θ* = 36.5° is distinctly visible, indicating that the MoSe_2_ sheets are not oriented perpendicularly to the substrate. This observation provides an explanation for the spontaneous peeling-off of the CIGSe/MoSe_2_ films from the Mo-coated SLG in some of the samples. All samples exhibit a shift of the MoSe_2_ (103) peak to lower angles. See Fig. 6b inset for a close-up of the (103) reflection indicating residual stresses and substrate-induced strain that could be caused by mechanical stress during the simultaneous peeling off of the CIGSe absorber layer due to high-temperature annealing and further intentional exfoliation procedure of the layer. The XRD pattern of the sample with ~ 600 nm of MoNa layer shows the strongest MoSe_2_ (103) diffraction peak while the weakest diffraction peak is observed in the sample with thinnest (~ 200 nm) MoNa layer. This suggests that a higher number of Mo atoms changes to MoSe_2_ in the presence of sodium (Jia and Zhou 2023). Estimation of global karst carbon sinks from 1950 to 2050s using response surface methodology, Geo-spatial Information Science. 10.1080/10095020.2023.2165974). The catalytic effect of Na precursor on the Se-diffused Mo has also been reported in previous studies (Rostan et al. 2005). Using the XRD data, Mo, CIGSe, and MoSe_2_ compounds are assessed in terms crystallite lattice configurations and possible defects. The Lattice constant ($\alpha$) is derived from Vegard’s law and Bragg’s law was employed to compute the structural variables [${d}_{hkl}=\left(\frac{n\lambda }{2{\text{sin}}\theta }\right)$] (Doroody et al. 2021). Crystallite diameter (${D}_{hkl}$) was estimated from the major (110) peak by Scherrer’s formula [${D}_{hkl}=0.9\lambda /\beta {\text{cos}}\theta$] while $\beta$ presents the Full Width at Half Max (FWHM) and $\theta$ as the Bragg’s angle at ($hkl$) plane (Doroody et al. 2021). On the other hand, micro strains are seen in the XRD graph as a result of lattice defects and displacement that was checked by employing Stoke Wilson formula [$\varepsilon =\beta /4{\text{tan}}\theta$] (Wilson et al. 2006) while Williamson and Smallman’s [$\delta =n/{D}^{2}$] formula estimated the dislocation density (Doroody et al. 2021). Table 3 summarizes the projected parameters. Variation in crystallite size extracted from the main (103) peak of MoSe_2_ is also represented in Fig. 6b. Improved crystallite agglomeration was observed to be linked to higher Na content, which resulted in lattice expansion in samples with the MoNa layer. Crystallite diameters from 20 to 30 nm are consistent with those described in other studies (Neugebohrn et al. 2017).

**Table 3:** Structural measures on sputtered Mo thin films

Sample	$hkl$	$\theta$	$\beta$(deg)	${a}_{{\text{cubic}}}$(Å)	${d}_{hkl}$(Å)	$D$(nm)	$\varepsilon$(× 10^−3^)
CIGSe/Mo	(110) Mo	20.31	0.37	3.14	0.222	24.25	4.30
CIGSe/Mo/MoNa1/Mo/SiO_2_	(110) Mo	20.28	0.32	3.14	0.222	27.25	3.83
CIGSe/Mo/MoNa2/Mo/SiO_2_	(110) Mo	20.23	0.30	3.15	0.223	29.36	3.57
CIGSe/Mo/MoNa3/Mo/SiO_2_	(110) Mo	20.25	0.30	3.15	0.223	29.55	3.54
Sample	$hkl$	$\theta$	$\beta$(deg)	${a}_{{\text{hexagonal}}}$(Å)	${d}_{hkl}$(Å)	$D$(nm)	$\varepsilon$(× 10^−3^)
CIGSe/Mo	(103) MoSe_2_	18.25	0.35	7.78	0.246	25.20	4.59
CIGSe/Mo/MoNa1/Mo/SiO_2_	(103) MoSe_2_	18.24	0.28	7.78	0.246	30.76	3.76
CIGSe/Mo/MoNa2/Mo/SiO_2_	(103) MoSe_2_	18.21	0.27	7.79	0.246	32.67	3.55
CIGSe/Mo/MoNa3/Mo/SiO_2_	(103) MoSe_2_	18.24	0.25	7.78	0.246	35.64	3.25
Sample	$hkl$	$\theta$	$\beta$(deg)	${a}_{{\text{tetragonal}}}$(Å)	${d}_{hkl}$(Å)	$D$(nm)	$\varepsilon$(× 10^−3^)
CIGSe/Mo	(112) CIGSe	13.44	0.63	8.12	0.331	13.61	11.44
CIGSe/Mo/MoNa1/Mo/SiO_2_	(112) CIGSe	13.53	0.49	8.06	0.329	17.40	8.89
CIGSe/Mo/MoNa2/Mo/SiO_2_	(112) CIGSe	13.47	0.56	8.10	0.331	15.31	10.15
CIGSe/Mo/MoNa3/Mo/SiO_2_	(112) CIGSe	13.47	0.54	8.10	0.331	15.79	9.84

Raman scattering measurements were performed on the exposed side to clarify the chemical properties and phase formation at this back interface and to confirm the behavior portrayed by XRD analysis. The samples with similar absorber growth condition analyzed by Raman spectroscopy stem and results were produced after Mo layer deposition.

**Fig. 7:**
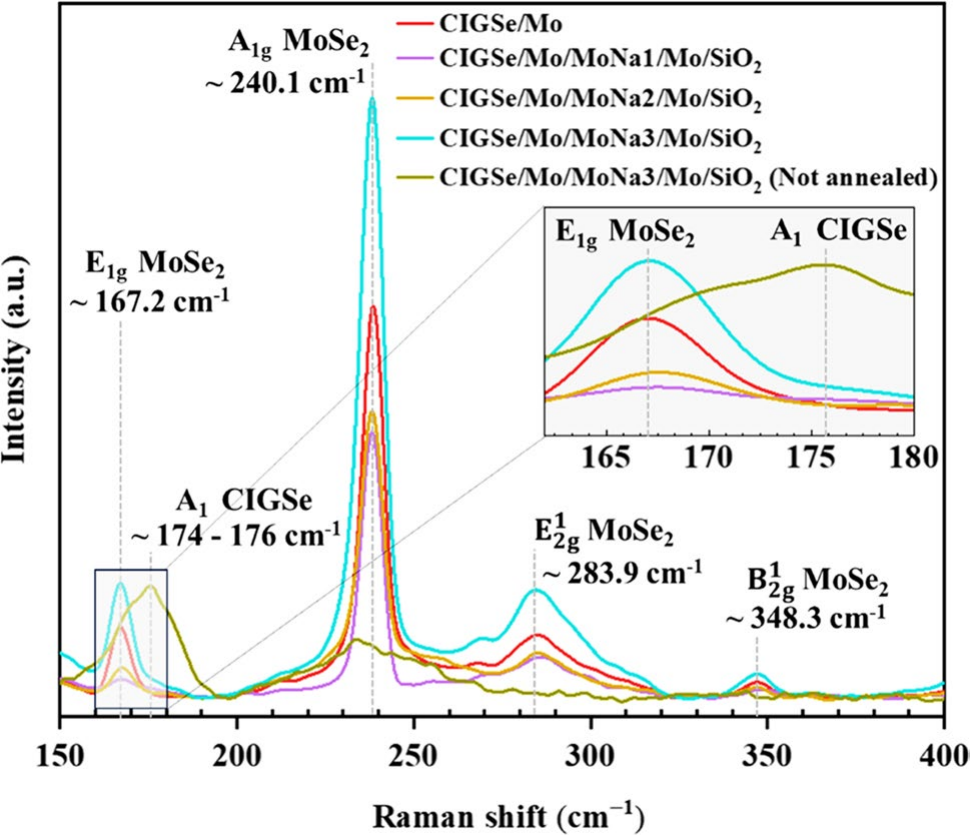
Raman graphs measured on exposed MoSe_2_/Mo surface

Figure 7 shows Raman spectra measured at the exposed MoSe_2_/Mo side of all samples. All spectra from the MoSe_2_/Mo showed highly intense ${A}_{1g}$ vibrational mode of MoSe_2_ at roughly 240.1 cm^−1^ which corresponds to the out-plane vibrational pattern of Se atom (Sugai and Ueda 1982). Another significant Raman mode to confirm the MoSe_2_ formation is observed at 167.2 cm^−1^, 283.9 cm^−1^, and 348.3 cm^−1^ assigned to the ${E}_{1g}$, ${E}_{2g}^{1}$, and ${B}_{2g}^{1}$ vibrational modes of 2H-MoSe_2_ compound, as reported by Sekine et al. (2013). For comparative purposes, Raman spectral measurement was conducted on unannealed sample with a 600 nm MoNa layer (at the MoSe_2_/Mo side). In contrast to the other spectra, this sample’s Raman spectra do not exhibit any prominent peaks indicative of the MoSe_2_ compound. Instead, the spectra are characterized by a broad band at around 174–176 cm^−1^, which is attributed to the A_1_ mode of the A^I^B^III^C^VI^_2_ chalcopyrite CIGSe compounds (Roy et al. 2002). The observation underscores that the reaction between Mo and Se, leading to the formation of the MoSe_2_ compound, can only take place when heat treatment is applied following the deposition of CIGSe film. Interestingly, the intensity of both Raman and XRD peaks associated with the MoSe_2_ compound notably increases with the rise in MoNa thickness, from 200 to 600 nm. The higher intensity of these peaks in the CIGSe/Mo/MoNa2/Mo/SiO_2_ sample indicates a more substantial presence of MoSe_2_, which can be indicative of a thicker MoSe_2_ layer.

**Fig. 8:**
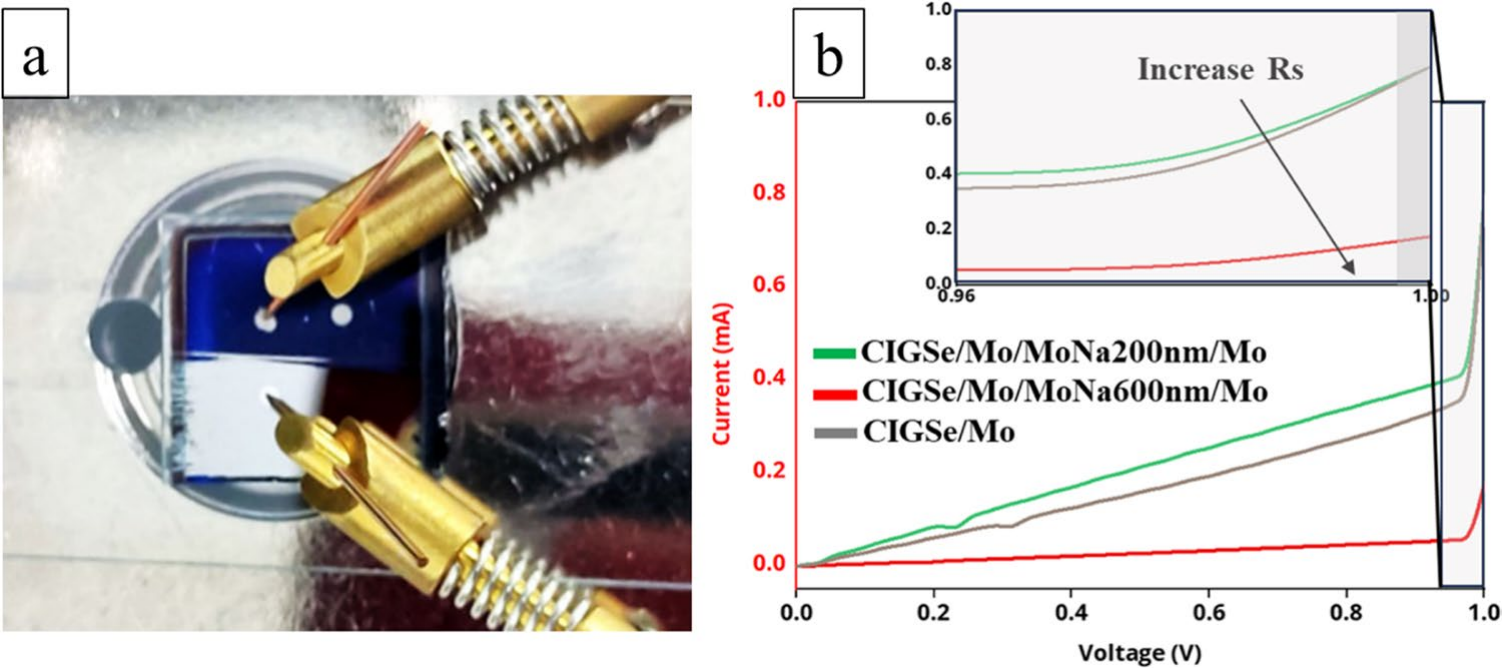
**a** Pin positioning condition for dark I–V; **b** dark I–V graph

To validate the aforementioned assumption, the standard setup in 25 °C has been utilized to measure I–V curves of the proposed stack as Fig. 8b shows. The IV measurement in the dark has the exceptional advantage of providing essential information on the properties of the CIGSe/Mo heterostructure, such as junction quality and contact resistances when the device is not in use (Soni et al. 2019). For electrical characterization, Ni contacts were deposited on samples and a photograph of one of the prepared samples engaged in a dark I–V measurement can be seen in Fig. 8a. The dark I–V curves for the samples have the diode characteristic as a p–n junction and displayed that while MoNa increased, the angle of descent at the forward bias dropped. Layer thickness is from ~ 200 to ~ 600 nm. I–V graph indicates that the hetero CIGSe/Mo with an overrange thickness MoSe2 layer is not as ohmic, but rather a Schottky connection (Yoon et al. 2014).

The leakage current ratio calculated from the saturation current decreases when the Na dopant in Mo thin films increases, yet the photovoltaic performance does not alter much. The resulting enhancement in junction quality revealed that Na incorporation from Na-doped Mo enhanced the junction properties of CIGSe cells (Kim et al. 2021). The Mo resistance increased as the thickness of the MoSe_2_ layer changed from 42 to 82 nm in the samples with a 600 nm thick of MoNa layer which was higher than that produced in the sample without the MoNa layer as shown in Fig. 8b, with mean values of 10 Ω and 13 Ω, respectively. The conductivity of proposed stack reduced with the increase in MoSe_2_ layer thickness. It pertains the MoSe_2_’s natural properties, whose resistivity is higher than Mo (Abou-Ras et al. 2005a, b, c; Zhu et al. 2012a, b). These results imply that optimal condition of the MoSe_2_ formation at the CIGSe/Mo interface can boost the CIGSe/Mo hetero-contact, from Schottky to ohmic contact. Ultimately, the significance of optimal Na ratio inclusion through alternative resources (MoNa) other than the SLG and its effect on CIGSe device performance is addressed with regard to optimal MoSe_2_ formation. Other strategies to optimize contact performance in CIGSe solar cells driven by Na inclusion involve the integration of a wide range of suitable substrates, the incorporation of advanced buffer layers to prevent impurity diffusion from the substrate (Li et al. 2017; Suresh and Uhl 2021), the introduction of new contact compositions with improved stability (Fonoll-Rubio et al. 2022), and the use of enhanced doping materials (Cai et al. 2018; Thomere et al. 2023).

## Conclusion

This research demonstrates the influence of sodium-doped molybdenum (MoNa) on CIGSe/Mo structures, and MoSe_2_ interlayer formation impacts on device electrical conductivity. Employing a layered MoNa/Mo structure with a sputtered SiO_2_ diffusion barrier on a soda lime glass substrate, controlled sodium diffusion is achieved. Notably, samples with a 600-nm-thick MoNa layer exhibit the highest resistivity (73 μΩcm) and sheet resistance (0.45 Ω/square). MoSe_2_ interlayer formation is more pronounced in CIGSe/(Mo/MoNa (600 nm)/Mo) structures compared to baseline CIGSe/Mo, with the thickest MoSe_2_ layer observed at 82 nm. XRD results showed a predictable reduction in metallic-Mo phases, attributed to increased MoSe_2_ compound growth rates due to Na diffusion, and a shift is observed in MoSe_2_ (103) peak angles, indicating non-perpendicular orientation, providing insights into the spontaneous peeling-off phenomenon in some samples during high temperature ($\ge$ 550 $^\circ{\rm C}$) annealing. The sample with ~ 600-nm MoNa layer displays the strongest diffraction peak and biggest crystal size (about 30 nm), suggesting a higher conversion of Mo to MoSe_2_ in the presence of sodium. Raman spectra analysis of MoSe_2_/Mo structures reveals a highly intense A_1g_ vibrational mode at approximately 240.1 cm^−1^, corresponding to the out-plane vibrational pattern of Se atoms. Additional Raman modes at 167.2 cm^−1^, 283.9 cm^−1^, and 348.3 cm^−1^ confirm the formation of 2H-MoSe_2_ compound. Notably, dark I–V measurements highlight the dark I–V curves of CIGSe/Mo heterostructures exhibit p–n junction characteristics, revealing a decrease in the angle of descent at forward bias as the MoNa layer thickness increases. The leakage current ratio decreases with higher Na dopant concentrations in Mo thin films, enhancing the junction properties of CIGSe cells. The conductivity of the stack decreases with increasing MoSe_2_ layer thickness, emphasizing the importance of optimal MoSe_2_ formation for transitioning from Schottky to ohmic contact in CIGSe/Mo heterostructures. Ultimately, the study emphasizes the critical role of MoNa in influencing MoSe_2_ growth and subsequently improving hetero-contact, providing valuable insights for optimizing contact properties in thin-film solar cell production. More research is recommended to study the impact of hypothetical back contacts with Na-control characteristics on Na diffusion from diverse sources.
